# Advances in Facial Composite Technology, Utilizing Holistic Construction, Do Not Lead to an Increase in Eyewitness Misidentifications Compared to Older Feature-Based Systems

**DOI:** 10.3389/fpsyg.2019.01962

**Published:** 2019-08-28

**Authors:** Graham E. Pike, Nicola A. Brace, Jim Turner, Hayley Ness, Annelies Vredeveldt

**Affiliations:** ^1^School of Psychology and Counselling, The Open University, Milton Keynes, United Kingdom; ^2^Department of Criminal Law and Criminology, Faculty of Law, VU University, Amsterdam, Netherlands

**Keywords:** facial composite, eyewitness identification, eyewitness memory, post-event information, unconscious transference

## Abstract

An eyewitness can contribute to a police investigation both by creating a composite image of the face of the perpetrator and by attempting to identify them during an identification procedure. This raises the potential issue that creating a composite of a perpetrator might then interfere with the subsequent identification of that perpetrator. Previous research exploring this issue has tended to use older feature-based composite systems, but the introduction of new holistic composite systems is an important development as they were designed to be a better match for human cognition and are likely to interact with memory in a different way. This issue was explored in the current experiment. Participants were randomly assigned to a feature-based composite construction condition (using E-FIT), a holistic-based composite construction condition (using EFIT-V) or a control condition. An ecologically valid delay between seeing a staged crime, creating the composite, and completing the identification task was employed to better match conditions in real investigations. The results showed that neither type of composite construction had an effect on participants’ accuracy on a subsequent identification task. This suggests that facial composite systems, including holistic systems, may not negatively impact subsequent eyewitness identification evidence.

## Introduction

The role that an eyewitness plays in a police investigation is likely to differ depending on whether or not the police are able to identify a suspect at a relatively early stage. If there is a suspect, then the eyewitness will usually be asked to participate in some form of identification procedure such as a lineup (commonly used in the US) or a video identification parade (commonly used in the UK). If the investigation has not yet generated a suspect, then the eyewitness might be asked to provide further assistance, including looking through albums of mug-shots (Dysart et al., 2001) or by creating a facial composite image (Brace et al., 2006).

The first generation of facial composite systems consisted of individual facial features that were printed on acetate and could be combined to form a face. Two such systems were Identikit, which used drawings of facial features, and Photofit, which used actual photographs. Toward the end of the 1980s, due to the increasing low cost and portability of personal computers, computerized systems such as E-FIT were introduced, which stored large image databases of facial features that could be combined using an interface that also allowed basic image manipulation. E-FIT construction begins by the operator interviewing the witness to gain a description of the perpetrator and then entering this into the system by selecting from lists of feature descriptors. An initial image, which is greyscale and fairly low resolution by contemporary standards, comprising the best match for the description provided is then displayed and shown to the witness, who attempts to improve it. This is done by the system displaying different features, always seen within the face, until the witness is happy that the particular feature is the best match possible. The system can also move and resize features; change skin, hair and eye tones; and add paraphernalia.

Research into the accuracy of the facial images produced by these feature-based systems found the images produced to be a generally poor likeness of the perpetrator (Laughery et al., 1977; Ellis et al., 1978; Laughery and Fowler, 1980). However, Brace et al. (2006) noted that this poor quality was not necessarily due to any limitations of the systems, as a skilled operator was able to produce a good likeness of a face he/she was familiar with, but rather had to do with the memory (particularly recall) and communication skills of the witness being insufficient for the task and not compatible with a feature-based approach. Making a comparable point, Wells et al. (2005) suggest that the task of composite building is an “unnatural act,” as it requires a witness to recognize and assemble a face from its component parts, a task that human cognition struggles with.

The move to a computer-based approach, such as E-FIT, meant that the witness always saw the individual features as being part of a whole face, rather than having to search through albums of individual facial features. However, construction still proceeded by selecting and manipulating individual facial features and so essentially used the same piecemeal approach as the earlier systems, an approach that had been shown to be problematic for witnesses (Pike et al., 2005). In an attempt to overcome this fundamental mismatch with human cognition, a new generation of composite construction systems was developed that not only involved showing a whole face to the witness, but utilized a holistic construction process as well (Tredoux et al., 2006; Frowd et al., 2007; Solomon et al., 2012).

One such “holistic” composite system is EFIT-V (branded as EFIT6 at the time of writing), which was the system used in the experiment reported in this paper. EFIT-V is based on a principal components analysis (PCA) approach (for a detailed technical description of how the EFIT-V system operates, see Gibson et al., 2003; Solomon et al., 2012). The basis of the system is that a database of faces is analyzed using PCA to determine a set of eigenfaces that describe the variation across the database. These eigenfaces can then be combined using different weightings in order to create any face within the “face space” described by the original faces in the database. If the database is suitably representative of the entire population of faces, then the system can be used to create any face. Using PCA means that the faces are stored in a holistic manner, i.e., a description of the entire face is used, rather than decomposing it into individual features, and that the construction process does not require the witness to work with individual features. EFIT-V uses an interface in which a grid of nine faces are presented to the witness, who selects the face they think is most similar to that of the perpetrator (although more complex selection tools are available, should the witness wish to do more than select the best match). The system then produces another grid of nine faces that resemble the face selected by the witness. The variation between the faces in the grid is gradually reduced: the first grid contains considerable variation, but by the 10th grid, the faces look far more similar to each other. In this way, the system uses information provided by the witness, through his/her selection of which face is the best match, to generate faces that should be successively more and more like that of the perpetrator.

This form of representation, presentation, and manipulation is entirely holistic, which is a good match for the way in which humans process faces (Laurence and Hole, 2012). Further, it is more based on recognition than recall, as the witness is not required to verbally describe the face at the start of the construction process, as was the case with older feature-based systems such as E-FIT. Indeed, systems using a holistic approach, such as E-FITV and EvoFIT (another holistic facial composite system utilizing a similar method to EFIT-V, but presenting grids of 18 greyscale facial images), were designed explicitly to be a good match for human cognition and to avoid a situation in which a witness is required to process a facial image in a way that would be “unnatural” (Gibson et al., 2003). Research has found that the holistic, PCA approach to composite construction does appear to be more compatible with human cognition (Brace et al., 2008) and, critically, to produce facial images that are a better likeness to the perpetrator (Frowd et al., 2010, 2012).

It is important to remember that composite images are used to help generate a possible suspect as part of a police investigation rather than a method of directly identifying the perpetrator. If the only evidence available is from an eyewitness, then generating suspects using the facial composites (and/or verbal descriptions) they provide may be the only way of progressing the investigation. If a suspect is generated, the witness may subsequently be asked to attempt to identify the perpetrator from a lineup or video parade in which the suspect appears. This means that a witness might produce a composite *and* be shown a lineup. It is therefore important to assess composite systems not just in terms of the accuracy of the facial image produced, but also in terms of whether they might interfere with the witness’ memory of the face to the extent that later identification from a lineup is affected.

Previous research has been conducted to explore what effect creating a composite, mostly using feature-based systems (e.g., Photofit or E-FIT), might have on subsequent eyewitness identification performance, and the results have not been consistent. Some studies have found that composite production interferes negatively with later identification accuracy (Wells et al., 2005; Topp-Manriquez et al., 2016), some that it does not have any significant effect (Yu and Geiselman, 1993; Davis et al., 2016; Pike et al., 2019), and some that it actually has a positive effect (Meissner and Brigham, 2001; Davis et al., 2014). Tredoux et al. (2016) conducted a meta-analysis of the research exploring the effect composite production might have on subsequent performance at an eyewitness identification procedure and concluded that creating a composite does not appear to have a statistically significant effect. However, many (52 out of 72) of the effect sizes included in the Tredoux et al. meta-analysis arose from experiments using older, feature-based composite systems or sketch artists.

Given the variability of results, it is worth considering in more detail the ways in which facial composites might interfere with memory. In explaining the impairment reported in target-present identification tasks, Wells et al. (2005) state that they “…strongly suspect that the impairment [to performance on the identification task] results from the process of having to break the face down into individual features to perform the composite building task” (p. 151). There is, of course, considerable support for the notion that faces are processed and remembered holistically, and not decomposed into their individual features (e.g., Baddeley, 1979; Tanaka and Farah, 1993; Wilford and Wells, 2010). This suggests that if witnesses were allowed to construct the composite in a more holistic manner, the impairment would have been reduced. In contrast, however, the notion that featural construction impairs memory for the target face is contradicted by findings that composites constructed in a featural manner can also improve subsequent face recognition (E-FIT; Davis et al., 2014; Identi-Kit; Mauldin and Laughery, 1981; free-hand drawing; McClure and Shaw, 2002).

An alternative explanation as to why composites might negatively impact later eyewitness identification accuracy relates to an observation by Mauldin and Laughery (1981), who suggest that more accurate or realistic representations of features “…may be more similar to the memory representation of the target face…” and therefore “…[a] significant amount of interference may result” (p. 356). Three studies have examined the role of similarity in more detail by varying the lineup images’ degree of similarity to the target face and the created composite (Comish, 1987; Kempen and Tredoux, 2012; Topp-Manriquez et al., 2016). In these studies, the target was always a composite face rather than a natural face to permit realistic morphing. All three studies found that participants’ memory for the composite they had created interfered with their memory for the target, although it is worth noting that the composites and targets were all composite faces, rather than real faces, so may have had a high degree of similarity to each other. However, a composite can also be similar to the target when the target is a real face and the composite itself also looks more like a real face than does a typical composite, as is the case when a modern, holistic system is used (e.g., Tredoux et al., 2006; Frowd et al., 2007; Gawrylowicz et al., 2012). Thus, on the one hand, we might expect less impairment when witnesses can construct the composite in a holistic manner, because it is a more natural process (Wells et al., 2005); on the other hand, we might expect more interference when the composite is created in a holistic manner, because it looks more realistic and is therefore more likely to interfere with the memory of the original face (Mauldin and Laughery, 1981). As part of the present paper, we provide a direct test of these competing hypotheses.

One study that has sought to answer this question was conducted by Davis et al. (2014), whose first experiment compared the influence of composite construction using E-FIT or EFIT-V on subsequent identification performance. They found that composite construction using either system resulted in improved identification performance compared to a no-construction control condition, but there was no significant difference between E-FIT and EFIT-V. In their second experiment, they again found a beneficial effect of EFIT-V composite construction (E-FIT was not tested), but in a follow-up study (Davis et al., 2016), there was no significant effect of EFIT-V construction. Although the work by Davis et al. was an important first step in assessing the influence of holistic composite construction, only one of their studies directly compared EFIT-V to E-FIT (i.e., a PCA-based to a feature-based composite process). Further, participants in all of their studies experienced only short delays between encoding and composite construction (0–30 min) and between composite construction and identification (5 min to 32 h). The current experiment was designed to compare the effects of composite building using a PCA-based system (EFIT-V) or a feature-based system (E-FIT) to a no-construction control condition, using delays more typical of real criminal investigations (Frowd et al., 2005), namely 2 days (mean 52 h) between encoding and composite construction and an average of 20 days between composite construction and identification.

## Materials and Methods

### Participants and Design

A total of 245 participants completed all three phases of the experiment. The data for a further 10 participants were excluded from analyses either because they did not attend all three stages or, in the case of two participants, because checks conducted at the end of the experiment indicated a chance that they may have previously seen the target. The participants (70% females, mean age 37.94, SD 12.76) were all working at or visiting the main campus of the Open University in a variety of capacities.

Participants were randomly assigned to one of six conditions of a 3 (Condition: E-FIT, EFIT-V, control) × 2 (Lineup: target-present, target-absent) factorial design. Participants were treated according to the ethical guidelines of The British Psychological Society and ethical clearance to conduct the experiment was granted by the human research ethics panel of The Open University.

This sample size was enough to detect a medium effect (of *φ* = 0.31 for the target-present condition and *φ* = 0.32 for the target-absent condition) with power = 0.80 at *α* = 0.05. This meant that the sample was sufficient to detect an effect considerably smaller than *φ* = 0.74 that was reported by Wells et al. (2005).

### Materials

Video sequences of staged crime scenarios were created for four targets, all of whom were Caucasian, aged approximately 30, and appeared without glasses or other paraphernalia. Two of the targets were male, with short, dark brown hair, and two female, with medium length, light brown hair. Each target was shown walking down a corridor while attempting to open the doors to a number of offices. When one door was found to be open, the target was seen entering the office, searching around, finding a mobile phone in an unattended jacket, placing it in his/her pocket and leaving the room. This sequence was edited to ensure that each target was viewed close-up for a minimum of 10 s as well as from a distance and from all angles. The edited sequences were 1 min 31 s, 1 min 50 s, 1 min 38 s, and 1 min 54 s in length for targets male 1, male 2, female 1, and female 2, respectively.

Composites were created by an operator using E-FIT (a feature-based composite system) or EFIT-V (a holistic, PCA-based composite system). The operator was a researcher trained in both composite systems. In the E-FIT condition, the participant’s verbal description of the target was used to generate an initial likeness. The participant then guided the operator in searching through and selecting alternative features and in moving and resizing these features. This process followed that used by police operators and witnesses in criminal investigations. In the EFIT-V condition, the participant’s verbal description was used only to select the appropriate database for sex and ethnicity. The EFIT-V system then displayed its first “generation” of nine randomly created face images from the eigenfaces within that sex and ethnicity set. The participant selected the “best match” from the face images displayed and the system then produced the next “generation” based on their selection. This continued until the participants were satisfied that they had produced as good a likeness as they could.

Although facial composite systems are used by law enforcement around the world, many of the systems were developed in the UK, where the standard is to use video identification procedures, not lineups. We decided to employ lineups in the current experiment so as to use a method more comparable to previous research in this area, most notably Wells et al. (2005). A target-absent and a target-present photo lineup were created for each target. The target-absent lineups consisted of nine foil images, while the target-present lineups consisted of eight foil images and an image of the target. The target’s image was a photograph taken separately (i.e., not a still from the crime video) and the lighting, background, clothing, and hair cues were different to those seen in the video. The foils for all lineups were selected by matching potential images to verbal descriptions of the targets provided by three participants who were unfamiliar with them. As part of the procedure for calibrating the lineups, three independent judges then checked each image to make sure that it was an approximate visual match for the target, for example to avoid foils containing any particularly distinctive features not mentioned in the verbal description. The foil images were sourced from the Pics image database maintained by Stirling University^1^. All of the images for the lineups were standardized so as to be pictorially similar (e.g., matched for size and resolution). The effective size of the lineup was measured using the technique advocated by Tredoux (1998). Sixty mock witnesses (who had not seen the targets) made a forced-choice selection from each lineup based on the modal description (derived from the descriptions provided by the three unfamiliar people). Analysis showed that all four lineups included at least seven plausible choices, with effective sizes for each lineup of 7.50, 8.57, 7.93, and 7.03. Analysis of lineup bias (see Malpass et al., 2007) revealed no statistically significant bias against the four targets, with exact probabilities of 0.09, 0.13, 0.16, and 0.13. Although non-significant, the probability of bias for target 1 (0.093) was a little high and less than 0.1, but as well as being non-significant, two foils were selected more frequently by the mock witnesses, so the lineup was deemed to be unbiased (Malpass et al., 2007).

### Procedure

Participants completed the experiment individually. There were three phases to the experiment. In the first phase, participants were informed that they were about to act as witnesses and would be shown a short video of a staged “incident.” They were told that they would see the video once only and that after seeing the video they would be asked to return approximately 2 days later to “give evidence.”

Approximately 2 days later (mean 51.6 h, range 42.42–73.0), participants returned to the lab for the second phase. They were first asked how well they could remember the perpetrator on a scale ranging from 0 (no memory of the target) to 100 (perfect memory of the target). They were then asked to provide a verbal description of the person depicted in the video, writing down as much as they could from the video sequence including clothing, body, and facial details. After providing their description, the 81 participants in the control condition left while the remaining participants constructed a facial composite of the target (84 participants using E-FIT and 80 participants using EFIT-V). On average, the time taken to construct the E-FIT composites (mean 30.33 min, SD 8.47) was considerably longer than the time taken for the EFIT-V images (mean 17.62 min, SD 7.05). This was partly because E-FIT construction requires an additional, initial stage in which the verbal description is entered and partly because the grid-based selection used in EFIT-V was designed to be a more efficient interface, avoiding having to look through large numbers of individual features as is the case with E-FIT. After constructing the composite, participants were asked to rate (out of 10) how similar they thought their composite was to the target (based on their memory of the target’s face).

Between 11 and 35 days after the interview (*M* = 20.26, SD = 4.85), participants returned to complete the third phase of the experiment. The large variation in this delay resulted from the practicalities in finding a time when the participant was able to reattend. In this final session, participants were again asked to rate how well they could remember the target, after which 124 participants were shown a simultaneous, target-present lineup and 121 a target-absent lineup. All were informed that the target may or may not be present in the lineup. The participant first indicated whether or not the target was present. If they thought the target was present, they indicated which lineup member they thought was the target. In either case, they were asked for a confidence rating from 0 “completely guessing” to 100 “completely certain.” Finally, participants were thanked and fully debriefed.

The facial composites that were produced by the participants were shown to three people who were familiar with the four targets employed in the study, who were asked to rate (out of 10) the similarity of each composite to the target. The raters judged similarity by comparing each composite image to their memory of the target’s face, rather than being provided with an image of the target. Memorial judgments were employed as previous research on composite construction has suggested that using a particular image can lead to matching specific pictorial elements between composite and photograph rather than face recognition *per se* (Brace et al., 2006) and because in a police investigation the hope is that someone familiar with the suspect will recognize the composite image, rather than compare the composite to a picture. The most forensically relevant method of evaluating composites would be to use spontaneous naming, as this would reflect how composites are used in a real investigation. While possessing ecological validity, spontaneous naming tends to lead to floor effects and is difficult to operationalize because priming effects mean a participant can only see one composite. However, it is important to note that ratings are a proxy for real-world naming and are not as ecologically valid.

## Results

As stated previously, of the 255 participants tested, data from 245 were included in subsequent analyses. To determine whether the delays between viewing the crime and constructing the composite/verbal description, or between constructing the composite and viewing the lineup, differed between conditions, 3 (Condition) × 2 (Lineup: target-present, target-absent) ANOVAs were conducted using the lengths of the two delays as the dependent variables. The results showed no statistically significant main effects or interactions, with $\eta_{p}^{2}$ values no larger than 0.016. In addition, logistic regression analysis revealed non-significant associations using lineup accuracy (whether a correct or incorrect decision was made) as the DV and the delays between crime and composite construction/description, *B = −*0.001, *p* = 0.99, and composite construction/description and lineup, *B = −*0.261, *p* = 0.34, as predictor variables.

Summary data of lineup outcomes are presented in Table 1, using the signal detection outcomes of hits, false alarms, misses, and correct rejections.

**Table 1:** Lineup outcomes by condition.

	E-FIT	EFIT-V	Control
**Target-present**	*N* = 43	*N* = 40	*N* = 41
Hit	28 0.65 (0.50, 0.78)	29 0.73 (0.57, 0.84)	25 0.61 (0.46, 0.74)
False alarm	10 0.23 (0.13, 0.38)	7 0.18 (0.09, 0.32)	11 0.27 (0.16, 0.42)
Miss	5 0.12 (0.05, 0.24)	4 0.10 (0.04, 0.23)	5 0.12 (0.05, 0.26)
**Target-absent**	*N* = 41	*N* = 40	*N* = 40
Correct reject	25 0.61 (0.46, 0.74)	26 0.65 (0.50, 0.78)	23 0.58 (0.42, 0.71)
False alarm	16 0.39 (0.26, 0.54)	14 0.35 (0.22, 0.50)	17 0.43 (0.29, 0.58)

*First row in each cell is the count, and second row is the proportion with 95% CI in parentheses*.

The data in Table 1 show that most participants (in all conditions) made a correct identification from the target-present lineup. Analysis of these data was based on the association between condition and the outcome of the lineup: where outcome was a “hit,” “miss,” or “false alarm” for target-present lineups, or a “correct rejection” or “false alarm” for target-absent lineups. A 3 (Condition) × 3 (Outcome) chi-square test conducted on the target-present lineup data revealed a statistically non-significant result with a small effect size, *χ*^2^(4) = 1.3, *p* = 0.86, *ϕ*_c_ = 0.07. Table 1 also shows that most participants (in all conditions) correctly rejected the target-absent lineup. A 3 (Condition) × 2 (Outcome) chi-square test on the data from the target-absent lineups revealed a statistically non-significant result with a small effect size, *χ*^2^(2) = 0.47, *p* = 0.79, *ϕ*_c_ = 0.06.

Although descriptive statistics (see Table 1) showed performance in the two composite conditions to be more accurate than the control condition (i.e., there were more hits for the target-present lineups and more correct rejections for the target-absent lineups), analyses of lineup outcome revealed non-significant differences, with small effect sizes between the E-FIT and control conditions for target-present lineups, *χ*^2^(2) = 1.7, *p* = 0.92, *ϕ*_c_ = 0.05, and target-absent lineups, *χ*^2^(1) = 0.10, *p* = 0.75, *ϕ*_c_ = 0.04, and for the EFIT-V and control conditions for target-present lineups, *χ*^2^(2) = 1.3, *p* = 0.53, *ϕ*_c_ = 0.13, and target-absent lineups, *χ*^2^(1) = 0.47, *p* = 0.49, *ϕ*_c_ = 0.08. The difference in lineup outcome between the E-FIT and EFIT-V conditions was also non-significant with small effect sizes for both target-present, *χ*^2^(2) = 0.55, *p* = 0.76, *ϕ*_c_ = 0.08, and target-absent lineups, *χ*^2^(1) = 0.14, *p* = 0.82, *ϕ*_c_ = 0.04.

To further explore the difference, specifically the lack of a difference, between the conditions in terms of lineup outcome, equivalence testing for categorical variables was performed employing the procedure recommended by Shiskina et al. (2018). This analysis, which is based on the Cramer’s V measure of effect size and follows the Beribisky et al., (2018, as cited in Shiskina et al., 2018) approach of setting the equivalence bound at *δ* = 0.3. As there is no direct method for computing the lower and upper confidence intervals in this instance, an iterative approach was taken to determine values for Δ*L* and Δ*U* (Smithson, 2003), using a function from the “DescTools” package for the R programming environment (Signorell et al., 2017). This procedure produced values for Δ*L* and Δ*U* for the condition by lineup outcome analysis in the target-present condition of (0.0, 0.13) and in the target-absent condition of (0.0, 0.20). In both cases, the upper bound falls below the equivalence bound of *δ* = 0.3, meaning that it is possible to conclude that the relationship between condition and lineup outcome is negligible for both target-present and target-absent lineups.

The quality of the composites produced by the participants in the E-FIT and EFIT-V conditions, as determined by the ratings of the independent judges, was compared to see if the images from one system were rated as being more like the targets than the other. Analysis revealed that the likeness ratings for the composites in the EFIT-V condition (*M* = 5.01, SD = 1.64) were significantly higher than those in the E-FIT condition (*M* = 4.11, SD = 1.36) with a medium effect size, *t*(118) = 3.29, *p* = 0.001, *d* = 0.60. The relationship between the likeness rating given to the composite constructed by a participant and that participant’s subsequent performance at the lineup is of interest because it might be that composite construction could impact memory differentially depending on how well the participant used the system. For example, the memory of participants who produced a poorly rated composite may be negatively affected, while the memory of those creating a highly rated composite might be improved. To test this relationship, logistic regression analysis was performed using whether the participant made a correct or incorrect decision at the lineup as the DV, and the mean likeness rating of the independent judges as the predictor variable, and revealed a non-significant association, *B* = 0.07, *p* = 0.54.

On average, the likeness ratings provided by the witnesses (*M* = 6.97, SD = 1.41) were higher than the mean ratings provided by the judges (*M* = 4.55, SD = 1.57), which a by-item analysis revealed as significant, *t*(119) = 13.9, *p* < 0.001, *d* = 1.61. There was also a statistically significant correlation between the two measures, *r*(120) = 0.19, *p* = 0.037, albeit with a relatively small effect size. Analysis of the witnesses’ ratings revealed the same pattern of results as for the judges’ ratings, though with a smaller effect size, with participants who created an EFIT-V providing higher ratings on average (*M* = 7.28, SD = 1.43) than did participants who created an E-FIT (*M* = 6.65, SD = 1.34), *t*(118) = 2.51, *p* = 0.014, *d* = 0.45.

The confidence data (see Table 2) were analyzed using a 3 (Condition: E-FIT, EFIT-V, control) × 2 (Lineup: target-present, target-absent) × 2 (Decision: correct, incorrect) ANOVA. This revealed a non-significant main effect of condition, *F*(2, 233) = 1.51, *p* = 0.22, $\eta_{p}^{2}$ = 0.01; a non-significant main effect of lineup type; *F*(1, 233) = 0.06, *p* = 0.81, $\eta_{p}^{2}$ = 0.00; and a significant main effect of decision accuracy (with higher confidence in correct than incorrect decisions), *F*(1, 233) = 49.98, *p* < 0.001, $\eta_{p}^{2}$ = 0.18. All of the two- and three-way interactions were non-significant.

**Table 2:** Mean confidence (0–100%) in identification decision.

	E-FIT	EFIT-V	Control	Total
**Target-present**	*N* = 43	*N* = 40	*N* = 41	
Hit	79.12 (16.95) [72.83, 85.38]	78.93 (16.07) [73.08, 84.78]	79.2 (17.36) [72.39, 86.01]	79.07 (16.56) [75.49, 82.66]
False alarm	71.0 (14.49) [62.02, 79.98]	40.0 (20.0) [25.18, 54.82]	60.91 (19.73) [49.25, 72.57]	59.29 (21.24) [51.42, 67.15]
Miss	58.0 (24.9) [36.17, 79.83]	57.5 (23.63) [34.34, 80.66]	40.0 (33.91) [10.28, 69.72]	51.43 (27.42) [37.07, 65.80]
**Target-absent**	*N* = 41	*N* = 40	*N* = 40	
Correct reject	71.2 (20.27) [63.25, 79.15]	73.45 (18.16) [66.29, 80.25]	76.09 (17.45) [68.96, 83.22]	73.45 (18.54) [69.22, 77.67]
False alarm	62.81 (18.35) [53.82, 71.80]	58.93 (21.32) [47.76, 70.10]	58.93 (24.86) [46.71, 70.35]	60.11 (21.38) [53.99, 66.22]
Total	71.43 (19.26) [67.31, 75.55]	69.11 (21.53) [64.39, 73.83]	69.07 (23.15) [64.03, 74.11]	69.89 (21.29) [67.23, 72.56]

*First row in each cell is the mean % with SD in parentheses, second is 95% CI in brackets*.

For participants who made a selection (i.e., who either chose the target or a foil) from the lineup, there were relatively strong and statistically significant positive confidence-accuracy correlations in the EFIT-V condition, *r_pb_*(50) = 0.57, *p* < 0.001, and in the control condition *r_pb_*(53) = 0.44, *p* < 0.001, and a weaker, but significant, correlation in the E-FIT condition, *r_pb_*(54) = 0.37, *p* = 0.007. The correlation coefficient in the control condition did not differ significantly from the correlation coefficients in either the EFIT-V condition, *Fisher’s Z* = 0.86, *p* = 0.39, or the E-FIT condition, *Fisher’s Z* = 0.42, *p* = 0.67, and the difference between the EFIT-V and E-FIT conditions was also non-significant, *Fisher’s Z* = 1.28, *p* = 0.20. For participants who did *not* make a selection from the lineup, there was a relatively strong and statistically significant positive confidence-accuracy correlations in the control condition, *r_pb_*(28) = 0.57, *p* = 0.002, and non-significant correlations in the E-FIT-V, *r_pb_*(30) = 0.28, *p* = 0.13 and the E-FIT condition, *r_pb_*(30) = 0.24, *p* = 0.21. However, the correlation coefficient in the control condition did not differ significantly from the correlation coefficients in either the EFIT-V condition, *Fisher’s Z* = 1.30, *p* = 0.19, or the E-FIT condition, *Fisher’s Z* = 1.45, *p* = 0.15, and the difference between the EFIT-V and E-FIT conditions was also non-significant, *Fisher’s Z* = 0.16, *p* = 0.87.

## Discussion

The results of the current experiment showed no detrimental *or* beneficial effect of constructing a facial composite compared to giving a verbal description only, regardless of whether a feature-based system (E-FIT) or a PCA-based system (EFIT-V) was used to construct the composite. Overall, participants tended to perform accurately on the identification task, with between 57.5 and 72.5% making a correct decision. Although these figures are a little higher than those reported in some eyewitness identification research, particularly after the long delay involved, the false alarm rates (which varied from 17.5 to 42.5%) are a good match for those in previous research and estimates for misidentifications in real investigations (Pike and Clark, 2018). This suggests that there were no floor or ceiling effects and that identification rates were at a level that would have reflected either a detrimental effect of composite production, resulting in a reduction in the identification rate compared to the control condition, or a beneficial effect, whereby identification rates would have increased compared to the control condition.

The results, therefore, revealed that composite construction using either a featural or holistic composite system did not have an adverse or beneficial effect on subsequent identification accuracy, using methods and materials that were a “fair” test of face memory (non-biased constructions and instructions were employed) and a good match for the procedures and delays involved in a real investigation.

These results differ from those reported by Davis et al. (2014), who found that creating either an E-FIT or EFIT-V image led to more accurate performance at the subsequent identification task. The likely explanation of this difference is that Davis et al. used relatively short delays between the initial exposure to the target face and composite construction (from 0 to 30 min) and then between construction and the identification task (5 min to 32 h), while the current experiment employed more ecologically valid delays of 2 days and 11–35 days. This explanation is supported by Mauldin and Laughery (1981), who reported that the initial beneficial effect of creating a composite on subsequent identification diminished as the delay between construction and identification increased.

The current results are similar to those of Davis et al. (2016), who also found that composite construction using a holistic system did not have an effect on later identification. As noted by Tredoux et al. (2016), individual studies of possible composite interference effects do show some variability, with only one outlier article showing a strong, negative relationship between composite construction and identification (Wells et al., 2005), although it should be noted that other research (Topp-Manriquez et al., 2016) using the (piecemeal, feature-based) FACES composite system employed by Wells et al. has also reported a large negative effect. Moreover, although some studies show a marginal, if statistically significant, effect in either direction, the general picture that emerges is that creating a composite does not alter a witness’ memory of the target face sufficiently to impact later identification.

The results reported here found that the EFIT-V system tended to produce more accurate composites, as determined through ratings provided by independent judges. As was stated previously, ratings are only a proxy for the spontaneous naming that would be needed in a real investigation, so it does not necessarily follow that the EFIT-V images would have been more recognized than the E-FIT images. The images produced by EFIT-V are much more photo-realistic than those of E-FIT, which are not only lower resolution and greyscale, but do not integrate the individual facial features seamlessly. This difference in the quality of the image produced is partly intrinsic to the two systems of course, and not a factor that can be easily separated out from the underlying representations and construction techniques used to construct the facial image itself. It could be that the high quality of the EFIT-V images was a factor that contributed to their higher ratings, though equally it is possible that their more photo-realistic nature could have raised expectations regarding their accuracy, potentially leading to stricter judgments if the representation of the face was not so perfect. In the current experiment, the ratings for the images produced by the two systems were fairly similar, albeit statistically significantly different, and there was considerable overlap in the range of ratings for the E-FITs (1.33–7.33) and EFIT-Vs (1.67–8.33), although it is possible that the increase in the use of the higher ratings (e.g., >7) for the EFIT-V images was driven in part by their higher image quality.

Analyses of the quality of the composites produced (here achieved with independent likeness ratings), can also shed light on an interesting theoretical distinction between the piecemeal and holistic construction processes and their differential effects on memory. Holistic systems (such as EFIT-V and EvoFIT) were developed to overcome the flaws in their predecessor piecemeal systems (such as E-FIT) and, in so doing, create images that were a better match to the face of the suspect. However, if the quality of the composites produced by both types of system was essentially the same, then this would suggest that either the experimental conditions were insufficient to allow the effects of holistic construction to manifest, or alternatively that holistic construction does not lead to more accurate composites. In either case, an experiment where the likeness of piecemeal and holistic composites was not sufficiently different would also mean that the potential effects (whether these be positive or negative) of holistic construction on the memory of the participants for the face of the target would not be revealed. In other words, if holistic construction was insufficiently influential to impact composite quality, it could also be insufficiently influential to impact eyewitness memory for the face of the target. In the experiment reported here, the holistic composites were judged to be more like the targets than the piecemeal composites, meaning that the impact of using a holistic construction method was apparent. However, although the descriptive statistics did show a tendency for participants who had constructed a holistic composite to be more accurate at the lineup, suggesting perhaps that their memory of the target’s face had been enhanced, this difference was not statistically significant from the performance of either the participants who created a piecemeal composite or the control participants. This suggests that although the holistic construction method did lead to improved composites, any enhancement of the participants’ memories was too short-lived to impact subsequent identification of the target in the lineup. This conclusion is consistent with that of Mauldin and Laughery (1981), and the results of Davis et al. (2014) where a shorter delay between construction and lineup was used, and improvements to face memory were found.

Analyses of the likeness ratings can also contribute to choosing between two potential hypotheses regarding why holistic and piecemeal systems might affect eyewitness memory differentially. If holistic systems produce better images, then regardless of the technique used in construction it could be that memory would be more affected simply because the witness would be viewing an image more like that of the perpetrator, an explanation very similar to that suggested by Mauldin and Laughery (1981) in relation to piecemeal composites. This could either have an enhancing effect, resulting through rehearsal of the target face, or a negative effect because the similarity of the image is more likely to change the original memory trace. Alternatively, it could be that the holistic construction process itself had a greater effect on memory for the target than piecemeal construction, regardless of the quality of the resulting composite image. The results reported here, that holistic composites were judged on average to be more like the targets but lineup performance was the same across holistic, piecemeal, and control conditions, and particularly that likeness was not a predictor of lineup accuracy, provide evidence that the former hypothesis is unlikely to offer the best explanation. Instead, the fact that holistic composites were rated as being more like the targets than piecemeal composites provides evidence that holistic construction is more effective than piecemeal, suggesting further research on its impact on face memory is needed.

The participants in the EFIT-V condition also tended to rate the accuracy of their own composite more highly than did participants in the E-FIT condition. This result suggests that the holistic construction approach adopted in the EFIT-V system resulted in the participants thinking they had produced better quality composites than the feature-based approach used in E-FIT. Interestingly, even though there was an increase in the quality of the EFIT-V images, there was no subsequent impact on identification performance compared to either the E-FIT or control conditions. This suggests that although holistic construction may lead to subjective judgments that a better composite has been produced, this does not interfere with, or indeed enhance, long-term memory for the target face.

Although the current experiment employed methods that sought a degree of ecological validity in some respects, not every aspect of the methodology was an effective match for a police investigation. One difference was that while a witness seeing a crime is likely to experience a degree, even a very high degree, of stress, participants in an experiment will experience a relatively low, or indeed no, degree of stress. The ethical requirements of conducting research mean stressing a participant is problematic, so this difference between experimental and real-life conditions is a standard issue for eyewitness research (Lane and Houston, 2019). Deffenbacher et al. (2004) conducted a meta-analysis of the effects of stress on eyewitness recall (36 independent tests) and identification (27 independent tests), which provided considerable support for the hypothesis that both recall and identification are adversely affected by high levels of stress. Morgan et al. (2004) drew a similar conclusion in one of the few studies to have introduced a high level of stress to experimental participants, by employing soldiers who were asked to identify their interrogators after having taken part in either a high- or low-stress interrogation. Some studies have found contradictory findings; for example, Sauerland et al. (2016) found that stress had no robust, negative impact on identification performance in a study that measured salivary cortisol levels to control for the effectiveness of the stressor. In addition, Brace et al. (2009), who used a field test in which participant-witnesses attended identification procedures at a police station, found that differences in the stress self-reported by participants were not reflected in their scores on a standardized checklist.

As Brace et al. (2009) note, as well as the stress experienced at the encoding stage while witnessing the crime, the stress induced by the various aspects of police investigation at the retrieval stage is also an important factor. In the case of composite construction, police operators have reported that the apparent stress levels of the witness can change during construction, with some witnesses becoming notably anxious as the face begins to resemble that of the perpetrator (Clark et al., 2000), suggesting that the effects of stress on composite construction may be complex and significant, and certainly worth further exploration.

Although finding that composite systems could lead to an improvement in eyewitness identification evidence would have been a positive effect for law enforcement, in the circumstances of a real criminal investigation “no effect” is in many ways a positive result. “No effect” in this case suggests that the same witness can be asked both to create a composite of the perpetrator and attempt to identify that perpetrator in an identification procedure, without the former interfering with the latter. Pike et al. (2019) drew a similar conclusion but pointed out that the delay between composite creation and seeing a lineup is often much shorter in experimental work than in a real case, so that care needs to be taken in translating the results. Even though a relatively long delay was employed in the current study, in a real case, the delay may be longer still; so again, care is needed in translating the results to practice. It is also important to remember that the research reported here, like previous studies, involved constructing a composite fairly soon after seeing the “crime” and sometime before seeing a lineup, so the results do not apply to an investigation with a long delay between crime and composite construction and a relatively short interval between construction and lineup.

In addition to variations in delay and stress levels, when considering translation of the results reported here into a policing context, it is important to remember that the current study involves just a single experiment; so although the results showed that composite construction did not interfere with subsequent eyewitness identification accuracy, employing delays that match those likely in a real criminal investigation, these data alone are not grounds for making claims about policing practice.

## Data Availability

The datasets generated for this study are available on request to the corresponding author.

## Ethics Statement

The studies involving human participants were reviewed and approved by Human Research Ethics Committee, The Open University. The patients/participants provided their written informed consent to participate in this study.

## Author Contributions

GP, NB, and JT contributed to the conception and design of the study. GP and AV performed the statistical analysis. GP, JT, and AV wrote the first draft of the manuscript. GP, NB, JT, HN, and AV wrote sections of the manuscript. All authors contributed to manuscript revision, read and approved the submitted version.

### Conflict of Interest Statement

The authors declare that the research was conducted in the absence of any commercial or financial relationships that could be construed as a potential conflict of interest.

## Footnotes

^1^http://pics.stir.ac.uk
